# Membrane fibreuse épirétinienne prenant la papille et la macula

**DOI:** 10.11604/pamj.2016.24.336.9672

**Published:** 2016-08-30

**Authors:** Aniss Regragui, Raja Daoudi

**Affiliations:** 1 Service Ophtalmologie A, Hôpital des Spécialités CHU Avicenne, Rabat, Maroc

**Keywords:** Rétine, membrane fibreuse, traction vitreo maculaire, Fibrous membrane, vitreomacular traction, retina

## Image en médecine

La membrane fibreuse épi rétinienne peut être a l origine de plusieurs complications graves notamment une traction vitreomaculaire ou un décollement de rétine. Le cas suivant montre une membrane fibreuse s étalant a la macula et la papille sans symptômes cliniques. L’image suivante montre le Fond d oeil d un patient de 49 ans diabétique qui consulte pour correction optique l examen trouve une membrane fibreuse épiretinienne s étalant sur la papille et la macula sans symptômes cliniques. Une surveillence régulière est obligatoire à la recherche de complications graves notamment une traction vitreomaculaire ou un décollement de rétine.

**Figure 1:**
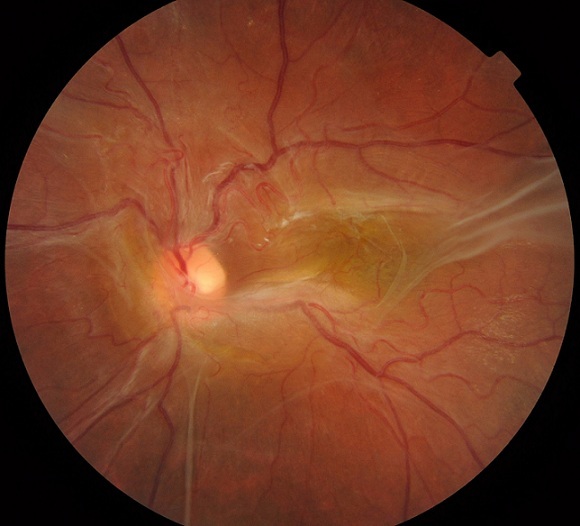
FO d’un patient de 49 ans qui présente une membrane fibreuse épirétinienne prenant la papille et la macula sans décollement de rétine ni traction vitréo rétinienne

